# Thermophoretic migration of vesicles depends on mean temperature and head group chemistry

**DOI:** 10.1038/ncomms15351

**Published:** 2017-05-17

**Authors:** Emma L. Talbot, Jurij Kotar, Lucia Parolini, Lorenzo Di Michele, Pietro Cicuta

**Affiliations:** 1 Department of Physics, Cavendish Laboratory, University of Cambridge, JJ Thomson Avenue, Cambridge CB3 0HE, UK

## Abstract

A number of colloidal systems, including polymers, proteins, micelles and hard spheres, have been studied in thermal gradients to observe and characterize their driven motion. Here we show experimentally the thermophoretic behaviour of unilamellar lipid vesicles, finding that mobility depends on the mean local temperature of the suspension and on the structure of the exposed polar lipid head groups. By tuning the temperature, vesicles can be directed towards hot or cold, forming a highly concentrated region. Binary mixtures of vesicles composed of different lipids can be segregated using thermophoresis, according to their head group. Our results demonstrate that thermophoresis enables robust and chemically specific directed motion of liposomes, which can be exploited in driven processes.

## Abstract (web summary)

Thermal gradients are shown to provide a robust and chemically specific driving force to liposomes. Here the authors show controlled direction of migration of unilamellar lipid vesicles by varying the temperature in the suspension and the exposed polar lipid head groups.

Thermophoretic drift of solid colloidal particles in response to a thermal gradient is capable of building up a significant concentration gradient^1^,^2^,^3^,^4^,^5^,^6^, and has led to a great deal of interest in thermophoresis as a separation and process engineering tool^7^. Thermophoresis of particles depends on the surface interactions with the solvent and on the size of the particle; the physical mechanisms are complex and not completely understood. In many cases, a high degree of particle accumulation has been achieved, with only marginal temperature differences (for example, <1 °C using thermal field-flow fractionation^8^,^9^,^10^,^11^). This concentrating effect can be very useful, for example, allowing the detection of quantities that would be unachievable in a dilute homogeneous sample, that is, thermophoresis can underpin a class of biological sensors^12^. Thermophoretic accumulation of lipids and nucleic acids in thermal vents has even been proposed as a key step in the origin of life, favouring the self-assembly of various essential components of life: nucleic acids^13^ and protocells^14^. In connection to this body of work, our results here add a further important piece to the puzzle, showing that also phospholipid vesicles are strongly thermophoretic, thus promoting conditions to form compartmentalized life. At much smaller scales, thermophoretic effects might be a relevant transport mechanism at play in and around biological cells, as compared to electrophoresis they are sensitive in different ways to ion concentration and hindrance from the cell membrane^15^. Okabe *et al*.^16^ found that the temperature difference between organelles can vary up to around 0.7 K, which could lead to thermal gradients of the order of 1 K μm^−1^. Due to a high sensitivity to changes in surface properties, thermophoresis has also proved valuable in measuring binding affinities and interaction strengths^17^.

There are two main attractive aspects of thermophoresis: directed motion, and separation of species. Directed motion lends the potential to transport a species to where it is needed via selective heating. This provides a potential strategy for drug delivery, or protein transport, and can enable the movement of encapsulated species that are inaccessible by other means, for example, within a vesicle. Fractionation of different species within a sample can be achieved by combining directed motion with concentration of a species to sort by size or composition. Most thermophoretic devices have focussed on separating particles by size to reduce polydispersity^18^,^19^,^20^, often in the context of constant surface properties. Current fractionation methods for separating lipid vesicles of varied composition rely on differences in density or surface charge: For example, ion-exchange columns allow charged vesicles (composed, for example, of phosphatidylglycerol (PG), phosphatidylserine (PS) or phosphatidic acid (PA) type head groups) to be fractionated^21^. Isopycnic centrifugation can be used to fractionate by density^18^,^22^, but care must be taken with osmotic gradients between the inner and outer vesicle medium, and timescales are often upwards of an hour. Thermophoresis has the significant benefit of not requiring a membrane nor other barrier to facilitate the separation; hence, there is great flexibility for continuous flow designs. Vesicle thermophoresis has not been studied previously.

Considering diffusion in combination with thermophoretic motion, the mass flux, *J*, can be expressed as


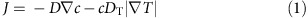


for dilute suspensions^23^, where *D* is the Brownian diffusion coefficient, *D*_T_ is the thermophoretic mobility, 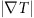 is the (assumed constant and uniform) temperature gradient and *c* is the concentration of the objects of interest. At steady state (*J*=0), and assuming a unidirectional vertical gradient along *z*, as is common in experiments (to avoid convection), equation (1) reduces to


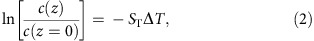


where *S*_T_ is the Soret coefficient, defined as


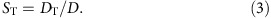


The direction of particle migration (towards hot or cold) depends on the sign of *S*_T_ and is system specific. For *S*_T_<0, migration is towards the hot side, whereas for *S*_T_>0, particles accumulate on the cold side. The magnitude of *S*_T_ sets the ratio of concentration on the hot side relative to the cold side at steady state.

Thermophoretic measurements on solid particles such as polystyrene beads^2^,^24^,^25^,^26^ have shown that many parameters (surface charge, particle size, particle–solvent interactions and particle concentration) all play a role in the migration direction and magnitude. A wide range of soft colloidal systems have also been explored, including polymers^5^,^27^,^28^, proteins^23^, DNA^6^,^29^,^30^, microgels^31^, micelles^32^ and microemulsions^33^,^34^. For all these systems, the magnitude of *D*_T_ is relatively unchanging, staying within the range 10^−7^–10^−8^ cm^2^ s^−1^ K^−1^, despite the large variation in surface chemistry and migrant size.

Vesicles are rich systems because they can be controlled in many ways: Unlike microemulsions, they are highly deformable, and the surface of a vesicle may behave as a solid or liquid depending on whether the temperature exceeds the melting temperature, *T*_m_, of the lipids comprising the vesicle (the so-called main chain transition temperature). Above *T*_m_, lipid diffusion rates in the bilayer are comparable to the diffusion rates within a liquid.

In this paper, we explore the temperature dependence of the Soret coefficient for vesicles with typical diameters of ∼1 μm, changing the surface chemistry of the lipid head groups with an aim to sort by composition. We show that the thermophoretic migration of vesicles can indeed be exploited for the directed concentration of vesicles composed of different lipid types.

## Results

### Thermophoresis of single-type lipid vesicles

**Figure 1:**
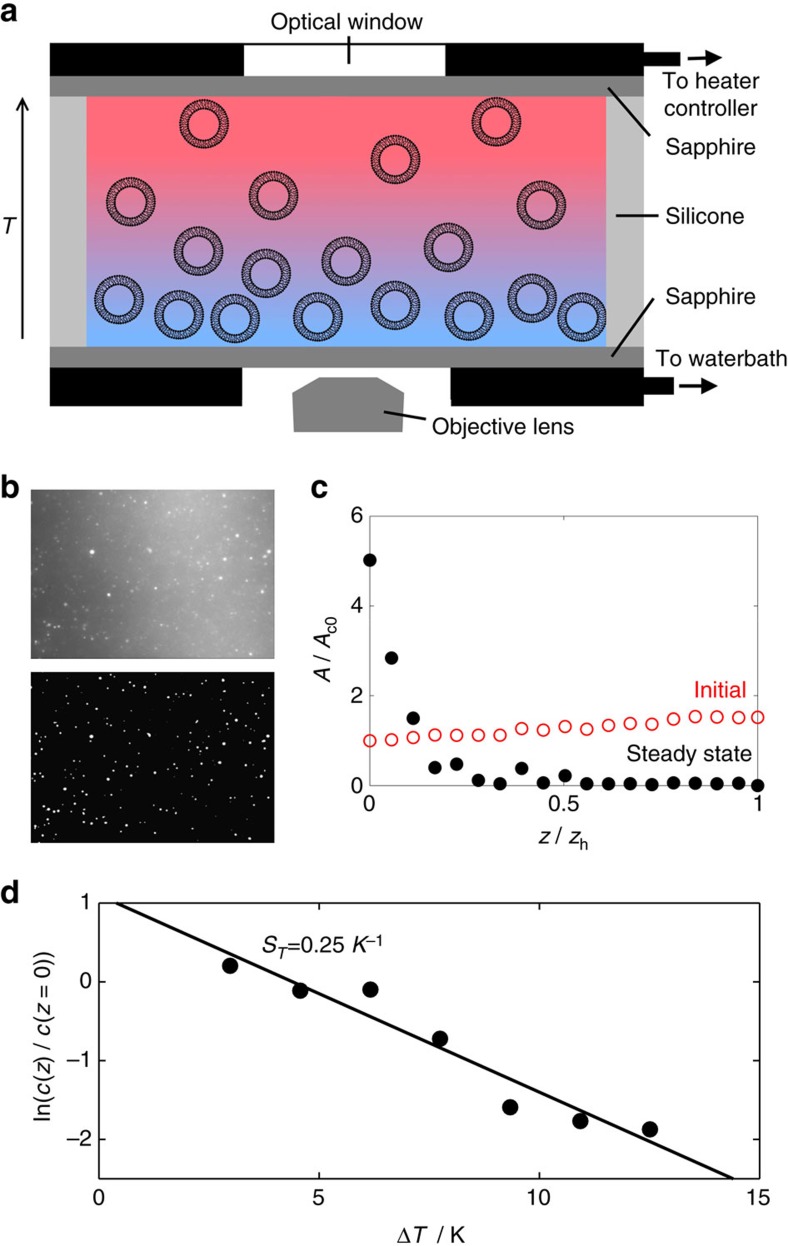
Vesicles respond strongly to thermal gradients. (**a**) Schematic cross-section of the custom-made temperature cell with a vertical thermal gradient (heating is from above). In this case, vesicles are thermophobic and move towards the lower (cold) sapphire window. (**b**) An example of a fluorescence image for a single slice and the corresponding binary conversion result obtained using MATLAB routines. (**c**) An example of the change in area fraction of vesicles in the slice, *A*, normalized by the initial area fraction at the cold plate, *A*_c0_, with height, *z*, above the cold plate (*z*=0) for thermophobic migration. The height is normalized by that of the hot plate, *z*_h_. (**d**) An example data set (DiPhyPC vesicles with *T*_hot_=45 °C and *T*_cold_=15 °C) showing at steady state an exponential concentration gradient with temperature (that is, also height), solid line fit on data, from which the value of the Soret coefficient *S*_T_ can be obtained as from the relation in equation (2).

**Figure 2:**
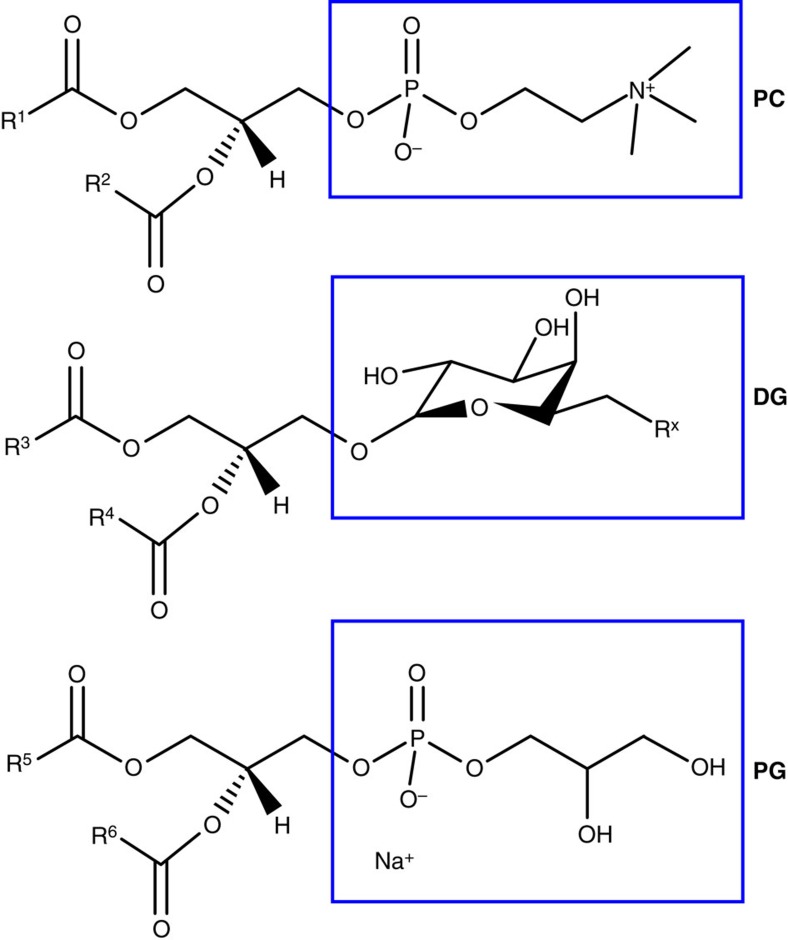
The chemical structure of lipid head group types PC, DG and PG. The box indicates *differences* in the head group structure between PC, DG and PG type head groups. R^*X*^ indicates any functional group.

We first explore thermophoresis in homogeneous samples of vesicles, using a custom-made temperature cell to generate a thermal gradient (Fig. 1a). The inclusion of a fluorescent lipid allows us to image the vesicle population throughout the cell (Fig. 1b,c). Lipids form bilayer membranes with their hydrophobic tail groups enclosed inside the bilayer, leaving their hydrophilic head groups exposed to the solvent; hence, we investigate the Soret coefficients of vesicles made up of lipids with a range of head group types. An example data set is given for 1,2-diphytanoyl-sn-glycero-phosphatidylcholine (DiPhyPC) in Fig. 1d. For the full range of lipids used in this paper and their abbreviated names, refer to the Methods section. For the structural differences between head group types: PC, DG, and PG, see Fig. 2.

**Figure 3:**
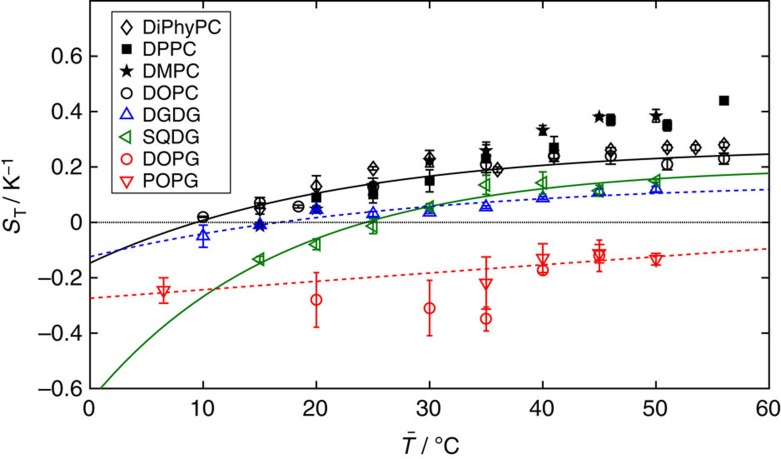
Soret coefficients depend on the lipid head group type and mean temperature. The Soret coefficients, *S*_T_, of 1 μm-diameter vesicles comprised of lipids with different head group chemistry (PC, PG, DG) are shown at various mean temperatures, with each head group set fitted by the empirical relation of equation (4); fit values are in Table 1. To avoid crowding, only one fit is shown for each different head group type (PC (*T*_m_<0), PG, DGDG, SQDG). Each colour represents a different head group type and each symbol a different tail group. Open symbols indicate *T*_m_≤0 and closed symbols *T*_m_>0. Error bars on data indicate the s.d. of *S*_T_ from five measurements taken at 5 min time intervals after achieving steady state. Note that all data points with the exception of 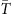
=5, 10 and 15 °C were obtained with 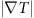
=0.15 K μm^−1^. The exceptions were measured at a lower 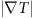 due to the minimum operational temperature of the water bath.

Figure 3 shows the variation in *S*_T_ with the mean temperature for 1 μm-diameter vesicles formed from a range of different lipids. Vesicles are of uniform lipid composition (that is, pure 1,2-dioleoyl-sn-glycero-3-phosphocholine (DOPC) or pure dipalmitoylphosphatidylcholine (DPPC). The dependence of *S*_T_ with mean temperature, 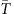, is well fit by the empirical relation described by Iacopini and Piazza^23^, which holds for a wide variety of hydrocolloids


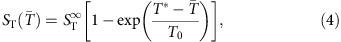


where 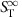 is the Soret coefficient in the high temperature limit, T* is the temperature at which *S*_T_=0 (that is, motion switches from thermophilic to thermophobic), and *T*_0_ indicates the magnitude of temperature effects. The Soret coefficients of vesicles are approximately an order of magnitude lower than those of polystyrene spheres of a similar size^2^ and are comparable to microemulsions with ∼15 nm radii^33^,^34^. Table 1 lists all the fit values in the paper, using equation (4).

The thermophoretic motion of vesicles can be grouped by their lipid head group composition. Vesicles composed of lipids with the same head group (for example, 1,2-dioleoyl-sn-glycero-3-phospho-(1′-rac-glycerol) (sodium salt) (DOPG) and 1-palmitoyl-2-oleoyl-sn-glycero-3-phospho-(1′-rac-glycerol) (sodium salt) (POPG)) have *S*_T_ values of the same sign and of similar magnitude at a given temperature (Fig. 3). The unexposed tail groups do not significantly influence *S*_T_. We conclude that variation in the magnitude and/or sign of *S*_T_ between different head group types is due to differences in the temperature-dependent interactions between the exposed head groups and the solvent. For example, changing the -R^*x*^ group (see Fig. 2) of the DG-type lipid (sulfoquinovosyldiacylglycerol (SQDG)’s sulfate group compared to digalactosyl diacylglycerol (DGDG)’s second sugar ring) dramatically alters *S*_T_ and T* despite large similarities in the remaining head group structure. The exception are vesicles composed of lipids that pass through their melting temperature within the observed temperature range (that is, those with *T*_m_>0). In this case, values of *S*_T_ for the same head group type are similar in sign and magnitude below *T*_m_, but on passing above *T*_m_ the magnitude of *S*_T_ increases above that of other lipid vesicles sharing the same head group (Fig. 3). Examples of this include DPPC (*T*_m_≳41 °C, ref. 35) and 1,2-dimyristoyl-sn-glycero-3-phosphocholine (DMPC) (*T*_m_≳23 °C, ref. 35), when compared to DiPhyPC (*T*_m_≳−120 °C, ref. 36) and DOPC (*T*_m_≳−20 °C, ref. 36).

For all head group types, with the exception of PG, thermophoretic motion accumulates vesicles on the cold side at temperatures ≳35 °C and on the hot side at 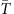
<10 °C. However, T* varies between head group types. Vesicles composed of DOPC, DiPhyPC, DMPC or DPPC are thermophobic until the mean temperature drops below ∼10–15 °C, whereas for vesicles of DGDG, the switch to thermophilic behaviour is at a higher mean temperature (∼17 °C). SQDG is a charged variation of the DG-type head group and switches to thermophobic behaviour at a higher mean temperature (∼24 °C) than its DGDG counterpart. POPG and DOPG vesicles are thermophilic over the entire observed temperature range. The enhanced thermophilicity of vesicles with a net charge (SQDG, POPG and DOPG) is likely due to the additional ionic contribution to *S*_T_. The negative shift in the *S*_T_ values of the charged lipids is consistent with the more thermophilic behaviour of negatively charged colloids resulting from the thermoelectric effect^37^. The higher surface charge density of the PG-type head group enhances the effect compared to the SQDG head group (same negative net charge but smaller surface area of the PG head group).

**Figure 4:**
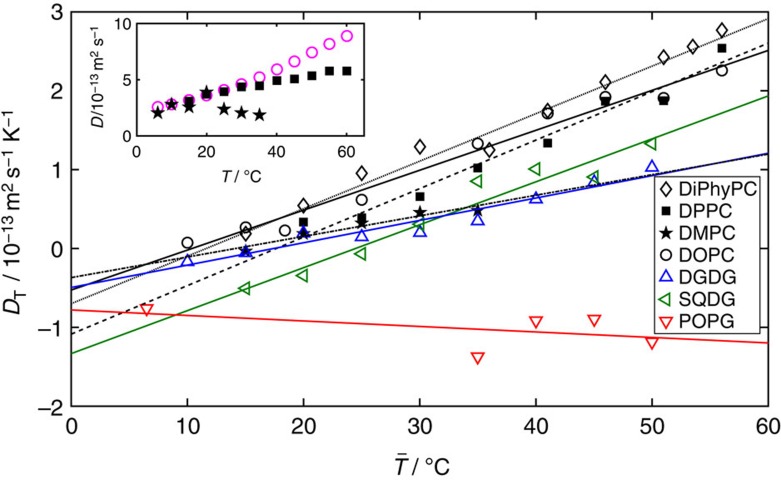
The thermophoretic mobility of vesicles depends linearly on temperature. Thermophoretic mobility, *D*_T_ (evaluated at 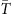), for lipids excluding the PG-type head group, increases linearly with mean temperature. The linear fits are used to measure values of *dD*_T_/*dT*, given in Table 1, and discussed in the text. Data are calculated from the measured Soret coefficients using equation (3), assuming a diffusion coefficient based on a sphere with a 1 μm diameter for lipids with negative melting temperatures and taking the temperature to be 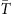. The measured diffusion coefficients (using differential dynamic microscopy^41^) for vesicles of lipids with non-negative melting temperatures are shown in the inset above and below the chain melting temperature (DPPC—*T*_m_≳41 °C (ref. 35) ◼) and DMPC—*T*_m_≳23 °C (ref. 35) (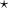)). A comparison is made with the expected diffusion coefficients (Stokes–Einstein) for spheres (○), where the viscosity dependence on temperature is accounted for.

For 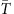
>35 °C, the variation in *S*_T_ for small Δ*T* is reduced (Fig. 3). SQDG vesicles exhibit the steepest change in *S*_T_ with decreasing temperature, followed by the PC-type head groups. The variation in *S*_T_ with temperature for POPG and DOPG vesicles is much lower than for the other lipid types. Figure 4 shows that indeed the thermophoretic mobility of lipids with the PG-type head group is nearly invariant with temperature. For all headgroups except the PG-type, d*D*_T_/d*T* ranges between 0.03 and 0.06 × 10^−13^ m^2^ s^−1^ K^−2^ (Fig. 4). The thermophoretic mobility of DPPC and DMPC increases linearly with temperature both above and below *T*_m_. There is no notable increase in d*D*_T_/d*T* above *T*_m_ for either DPPC or DMPC as the temperature is raised. Instead, the increase in the Soret coefficient (compared to that of DiPhyPC or DOPC) at temperatures exceeding *T*_m_ is induced by the decrease in the diffusion coefficient (see inset in Fig. 4), possibly resulting from shape changes of the vesicles above *T*_m_ away from spherical.

**Figure 5:**
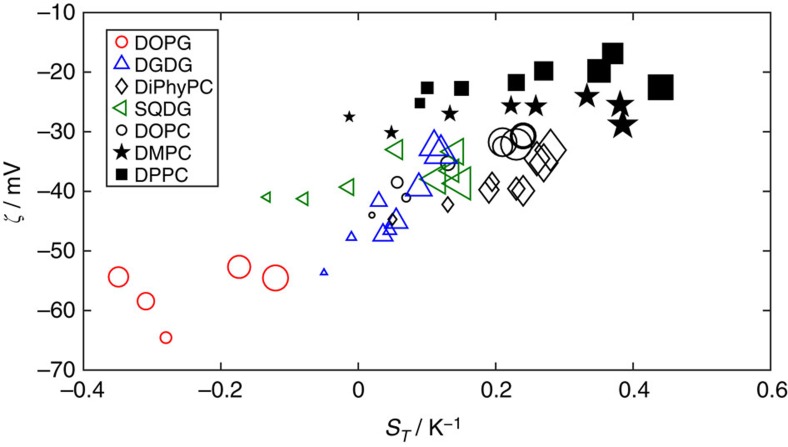
The Soret coefficient correlates with the vesicle zeta potential across lipid species. However, there is no correlation within each lipid type. Larger marker size indicates a higher measurement temperature. The smallest markers are for 10 °C up to the largest markers at 55 °C. For clarity, error bars for the data in Fig. 5 are given in Supplementary Fig. 1.

In order to rationalize the dependence of the Soret coefficient on different lipid head group types, comparisons were drawn between lipid size or vesicle *ζ*-potential. First, there is no correlation between lipid molecular weight and *S*_T_ (see Supplementary Fig. 2). Second, as can be seen in Fig. 5, there is no correlation for each separate lipid subspecies (for example, DOPC) between the *ζ*-potential of the vesicles (*T*-dependent, as in many systems) and the corresponding *S*_T_ over a range of temperatures (except for DGDG). This suggests that the *ζ*-potential itself does not underpin the *T* dependance of *S*_T_. However, across all lipid species there is a correlation of the *ζ*-potential with *S*_T_, with a more negative *ζ*-potential giving more thermophilic behaviour of the vesicles. This suggests that another common factor, such as general hydrophilic character (which would be proportional to the average surface charge) may be the root cause of correlations between *ζ*-potential and *S*_T_. The *ζ*-potential of a lipid may therefore give a prediction of thermophoretic response.

### Sorting vesicles by composition

By controlling the mean temperature between the plates, vesicles can be directed towards hot or cold. As the head group type influences T*, the direction of motion of vesicles composed of each head group type is controllable relative to the others. The difference in the sign and/or magnitude of *S*_T_ for vesicles comprising different lipids offers a thermophoretic method for separating vesicles according to head group composition. Here we explore thermophoretic migration in binary vesicle mixtures of DOPC/POPG and DOPC/DGDG, with the aim to see if separation by lipid head group type is indeed possible.

**Figure 6:**
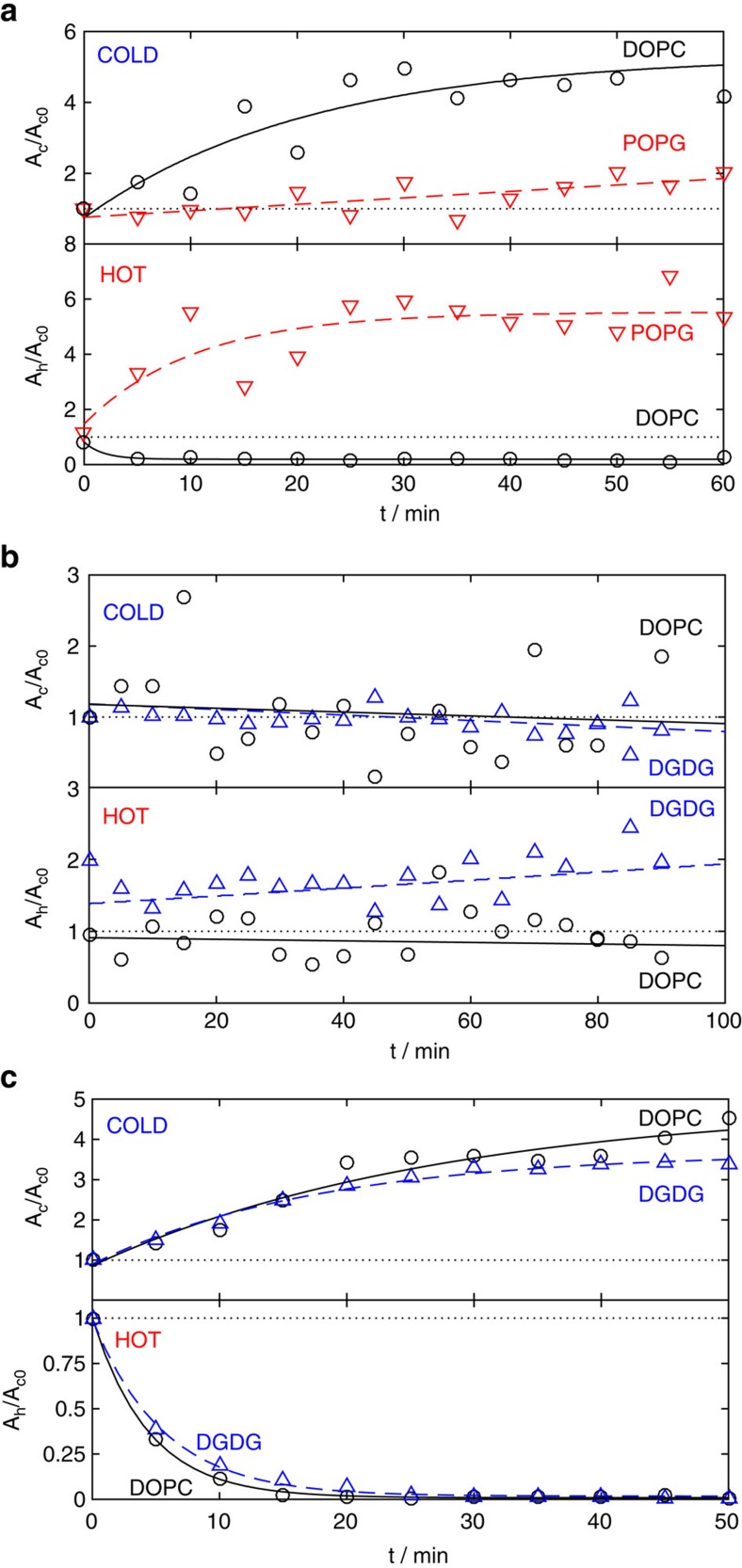
Vesicles can be segregated by composition. Examples of thermophoretic migration are given for a mixture of 1 μm vesicles of (**a**) POPG (dyed with Texas red) and DOPC (dyed with Oregon green) with *T*=25 °C at the cold plate and *T*=55 °C at the hot plate, (**b**) DGDG (dyed with Texas red) and DOPC (dyed with Oregon green) with *T*=5 °C at the cold plate and *T*=20 °C at the hot plate and (**c**) DGDG (dyed with Texas red) and DOPC (dyed with Oregon green) with *T*=35 °C at the cold plate and *T*=65 °C at the hot plate. The area fraction of each species at the cold plate, *A*_c_, and at the hot plate, *A*_h_, are shown with time, normalized by the initial area fraction at the cold plate, *A*_c0_. Dashed and solid lines are exponential fits to the data (fit parameters are given in Supplementary Table 1).

POPG vesicles exhibit thermophilic behaviour over the entire temperature range tested, while DOPC vesicles are thermophobic for 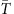
≳10 °C (Fig. 3). Separation is carried out at a mean temperature between the plates of 40 °C (Δ*T*=30 °C) to ensure a significant concentration difference between the plates for each vesicle type. This also results in Soret coefficients of comparable magnitude for both vesicle species. The opposite sign of *S*_T_ for POPG compared to DOPC enables significant separation of each species, with DOPC gathering on the cold plate and POPG gathering on the hot plate (Fig. 6a). While the centre of the cell becomes increasingly depleted of POPG with time, there is a slight increase in the POPG concentration on the cold side (see Supplementary Fig. 3). We attribute this small accumulation on the plate at low temperature to the attraction of POPG vesicles (which have a negative net charge) to the positively charged sapphire surface. The overall effect gives a separation of DOPC:POPG of 70:30 on the cold side and 4:96 on the hot side. DOPC gathers to the cold side (and depletes the hot side) at a faster rate than POPG, which can be quantified by the exponential fits to the data in Fig. 6 (fitting parameters are given in Supplementary Table 1).

There are two separation strategies for DOPC/DGDG vesicle mixtures, position the system in: (i) a temperature range where migration of DGDG vesicles is thermophilic but migration of DOPC vesicles is thermophobic (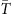
=12.5 °C, Δ*T*=15 °C); (ii) a temperature range where migration of both species is thermophobic but the Soret coefficients differ in magnitude (for example, 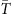
=50 °C, Δ*T*=30 °C).

Figure 6b shows the segregation of vesicles for case (i). DOPC is only marginally thermophobic in this temperature range; therefore, the concentration of DOPC at the hot and cold plates differs very little from the initial concentration. DGDG migrates to the hot side, giving a segregation on the hot plate of DOPC:DGDG of 24:76. On the cold side, there is little difference in the relative concentrations of each species. In comparison, approach (ii) results in similar thermophobic migration of both vesicle types, accumulating both species on the cold plate. The *S*_T_ value for DOPC is higher than for DGDG and so results in enhanced concentration of DOPC on the cold plate (compared to DGDG) at late times. The faster dynamics of DOPC compared to DGDG is also reflected in the exponential fitting parameters for the data in Fig. 6c (see Supplementary Table 1). The relative concentration of DOPC:DGDG on the cold side after 50 min was 57:43 (Fig. 6c). Due to depletion of both species away from higher temperatures, there is little difference in the relative concentrations on the hot side. The separation ratios indicate that in this case method (i) is more efficient at separating the two vesicle species.

For each of the binary vesicle mixtures, good separation by head group is achievable (upwards of three times the concentration of vesicle type 1 compared to type 2). For vesicles with *S*_T_ values of opposite sign and high magnitude (for example, POPG and DOPC), the segregation approaches ‘complete’ on the hot side (24 times vesicle type 1 to type 2). The technique could easily be applied to a wider mixture of head group types, for example, ternary or quaternary mixtures, and developed into a simple separation device with reasonably fast separation times (20–60 min). A continuous flow device similar to that developed by Piazza *et al*.^4^ would be appropriate, which used flow rates up to 0.05 μl min^−1^ and channel widths of 75 μm. Similar possibilities arise from this work, but a higher temperature gradient would be required to reach steady state after the same time frame. This separation method in the presence of flow has the potential for high-throughput sorting of cell blebs by composition and the fractionation of vesicles including or excluding particular membrane proteins.

### Vesicles of two lipid components and a sterol

**Figure 7:**
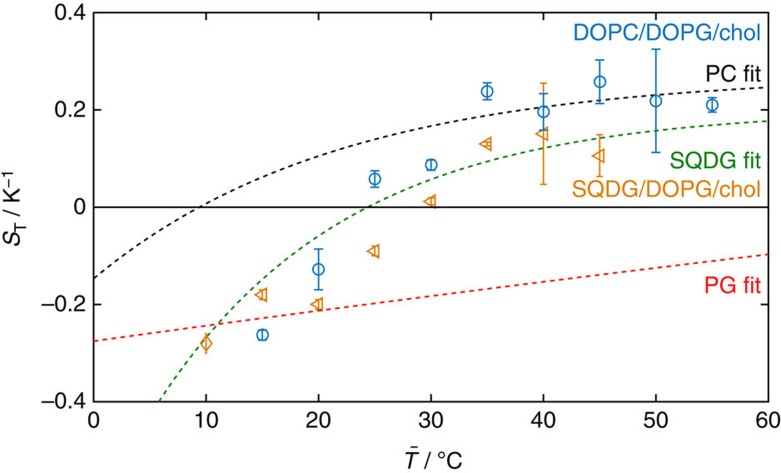
Thermophoretic properties can be designed by working with vesicles of mixed lipid composition. Here the Soret coefficients for vesicles of the ternary mixtures SQDG/DOPG/chol (27.5:27.5:45 mol%) and DOPC/DOPG/chol (27.5:27.5:45 mol%) are seen to transition between the limiting values for each lipid component (dashed lines), as the mean temperature is increased. At low temperatures, the Soret coefficients closely match the PG-type lipid. Error bars on data indicate the s.d. of *S*_T_ from five measurements taken at 5 min time intervals after achieving steady state.

**Figure 8:**
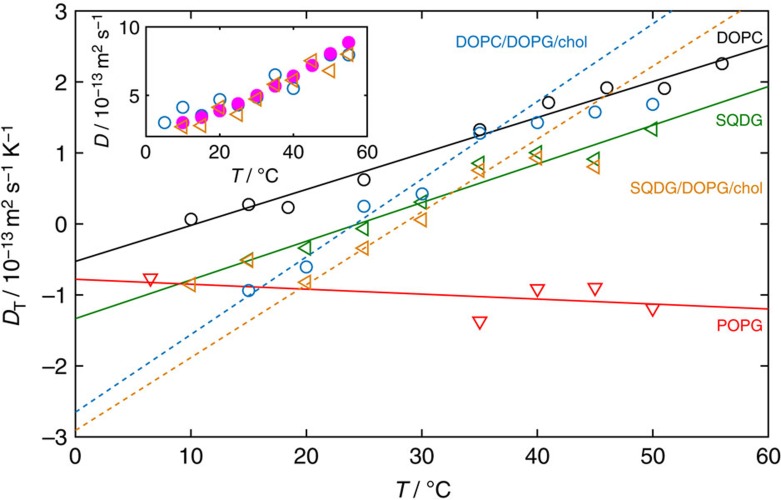
In mixed lipid vesicles the thermophoretic mobility depends more strongly on temperature. SQDG/DOPG/chol and DOPC/DOPG/chol mixtures have enhanced thermophoretic mobility compared to their single lipid counterparts for mid-range temperatures. Dashed lines indicate linear fits for the ternary mixtures in the transitional regime and solid lines for the single lipid vesicles. The steeper gradient, *dD*_T_/*dT*, in the transitional regime demonstrates the enhanced sensitivity of the thermophoretic mobility in this region. The measured diffusion coefficients (by differential dynamic microscopy) for the ternary mixtures are shown in the inset and closely match the expected diffusion coefficients for spheres of the same size (closed ○).

For comparison with the vesicles made of only a single lipid, we look at vesicles composed of two lipids and a sterol, where each component lipid provides an opposite response, that is, one is thermophilic and the other is thermophobic. For this purpose, we choose SQDG/DOPG/cholestanol (chol) and DOPC/DOPG/chol vesicles (with component ratios of 27.5:27.5:45 mol%). Note that vesicles formed from these compositions do not phase separate into micrometer-scale domains (we have not investigated if nanodomains form). The chol ensures membrane fluidity and inserts into the hydrophobic part of the bilayer so is unexposed to the solvent. As such, the chol has negligible effect on the Soret coefficient (Supplementary Fig. 4), as with the lipid tail groups. In this case, we see an interesting transitory behaviour, where at high mean temperatures *S*_T_ follows the curve for the more thermophobic component, and at low mean temperatures, *S*_T_ follows the curve of the more thermophilic component, shown in Fig. 7. At intermediate temperatures, the competing thermophoretic responses result in an intermediate *S*_T_. The change in *S*_T_ with temperature is increased compared to that of the single components near the transition from thermophilic to thermophobic behaviour (*S*_T_=0). The magnitude of *S*_T_ therefore changes more significantly over a smaller temperature range, enabling a switch from a high magnitude *S*_T_ of negative sign to a high magnitude *S*_T_ of positive sign. Indeed the variation in the thermophoretic mobility with temperature is enhanced during the transition, with d*D*_T_/d*T*∼0.10–0.11 × 10^−13^ (Fig. 8). The diffusion coefficients for the SQDG/DOPG/chol and DOPC/DOPG/chol mixtures closely match those expected for spheres (see inset in Fig. 8).

The enhanced thermal response has potential for use as a temperature sensor by including ternary vesicles (comprising component lipids with opposite thermophoretic responses) incorporating different dyes: In a manner similar to a Galileo thermometer, vesicles would then respond strongly to a gradient and result in a measurable optical change. For example, DOPG/DOPC/chol vesicles switch from thermophilic to thermophobic behaviour with *S*_T_ values of reasonable magnitude (0.1 K^−1^) between 20 °C and 25 °C and SQDG/DOPG/chol vesicles switch between 25 °C and 35 °C. If SQDG/DOPG/chol was dyed red and DOPC/DOPG/chol was dyed green, then as the mean temperature rises above ∼23 °C, the colour at the hot plate will change from yellow (red+green) to green.

The sharp change in the gradient of *S*_T_ with mean temperature seen in these ternary vesicles with opposing components is unusual—normally the gradient changes gradually with increasing/decreasing mean temperature following the empirical relation given in equation (4). The abrupt change is best seen in Fig. 7 at 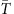
∼20 °C, where the thermophoretic behaviour changes from transitional to follow that of the DOPG vesicles. This behaviour contrasts with that of mixed micelles^1^, which only show a smooth change in *S*_T_ with 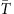, and intermediate behaviour between the two components (*S*_T_ values do not reach those of either component). However, there are similarities with poly(*N*-isopropylacrylamide) (PNIPAM) migrogels, which exhibit a sharp change in the magnitude of *S*_T_ after passing through the coil-to-globule transition temperature^31^. In this case, the change in *S*_T_ with temperature is also enhanced (compared to single PNIPAM chains).

## Discusion

Large (micron sized) unilamellar lipid vesicles migrate in a thermal gradient to accumulate either at hot or cold temperatures, resulting in significant concentration gradients. By tuning the mean temperature, the direction of the migration can be changed. The Soret coefficient, which characterizes the migration, depends on the chemical structure of the lipid head group. The charged lipids DOPG and POPG make vesicles thermophilic over a wide temperature range, whereas vesicles made from neutral lipids exhibit a switch from thermophilic to thermophobic behaviour above 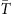
∼10–25 °C. For lipids with a melting temperature >0 °C, passing above the melting temperature results in an increased Soret coefficient, possibly as a consequence of shape changes away from a sphere.

Thermophoresis provides a powerful tool for separation of vesicles by their head group composition. Binary mixtures of vesicles can be successfully separated by setting the mean temperature to ensure thermophilic motion of one type of vesicle and thermophobic motion of the other. In particular, when the concentrating effect of vesicles is strong, and each vesicle species migrates to the opposite side of the cell, segregation ratios of 4:96 are possible; This is approaching complete separation. Separation is also possible when both vesicle species in a binary mixture are thermophobic at the separation temperature. However, the degree of separation is less than for the thermophilic/thermophobic population approach.

For ternary vesicles without domain formation and opposite thermophoretic responses of the two lipid components, the Soret coefficient transitions between the magnitude and sign of each of the components, tending towards the thermophilic component at low temperatures. The thermal response is enhanced during the transitional region, which has good potential for use in a temperature-sensing device.

The selective separation of vesicles demonstrated here could prove useful for directed delivery of phospholipid carriers with controlled surface chemistry that contain an encapsulated drug (independent of the drug surface chemistry). One can also envision selective transport of vesicles composed of desirable lipids budded from one membrane and directed to a second membrane to deliver those lipids. Furthermore, different lipid ratios result in a range of domain morphologies in vesicles^36^; sorting by composition could allow these morphologies to be separated. Strong phospholipid vesicle thermophoresis also seems significant in the context of conditions that would have favoured the origin of cellular life.

In principle, the many degrees of freedom of a vesicle (shape, fluidity of the bilayer, arrangements of lipids) could also give rise to a thermal response, making the behaviour different from solid particles or relatively undeformable liquid droplets, even for a given surface chemistry.

## Methods

### Reagents

The following lipids were purchased from Avanti Polar Lipids (Alabaster, AL) in chloroform: DOPC, DPPC, DMPC, DiPhyPC, DGDG, SQDG, DOPG, and POPG. Texas red 1,2-dihexadecanoyl-sn-glycero-3-phosphoethanolamine, triethylammonium salt (TX-DHPE) and Oregon green 1,2-dihexadecanoyl-sn-glycero-3-phosphoethanolamine (OG-DHPE) were purchased from Thermo-Fisher Scientific. Cholestanol (chol) was procured from Sigma-Aldrich for use in ternary mixtures. Chain-melting temperatures for the lipids are as follows: DOPC (*T*_m_≳−20 °C, ref. 36), DPPC (*T*_m_≳41 °C, ref. 35), DMPC (*T*_m_≳23 °C, ref. 35), DiPhyPC (*T*_m_≳−120 °C, ref. 36), DGDG (*T*_m_≳−50 °C, ref. 38), SQDG (*T*_m_≳−10–20 °C, ref. 38), DOPG (*T*_m_≳−18 °C, ref. 39), and POPG (*T*_m_≳−5 °C, ref. 40).

### Preparation of vesicles

Large unilamellar vesicles with ∼1 μm diameter were prepared by extrusion. First, 45 μl of a lipid solution in chloroform (4 mg ml^−1^) was deposited into a brown glass vial and dried to a film under vacuum. The solution included either TX-DHPE or OG-DHPE at 0.8 mol% to allow fluorescence imaging of the vesicles and to distinguish between vesicle mixtures. Second, the lipid film was rehydrated with 300 μl of water (MilliQ) and vortexed for 5 min. After transferring the sample to an Eppendorf tube, four freeze/thaw cycles were carried out in liquid nitrogen followed by water at 30 °C to facilitate break up and aid in forming a homogeneous large unilamellar vesicle suspension after extrusion. The sample was then extruded using a mini-extruder (Avanti Polar Lipids, Alabaster) with a polycarbonate track-etched membrane with 1.0 μm pores (Whatman). A typical size distribution for the vesicles formed via this method is provided in Supplementary Fig. 5. The initial concentration of vesicles in a sample is approximately 1% by volume.

### Thermal gradient and imaging

To measure *S*_T_, a vesicle suspension was loaded into a custom-made cell across which a vertical temperature gradient was applied with a temperature difference of Δ*T* and a mean temperature of 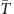 between the plates (see Fig. 1a). The applied temperature gradient was typically between 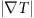
=0.05–0.15 K μm^−1^. The top of the cell was heated and the bottom cooled to avoid convection. The temperature of each plate was measured using thermocouples (precision approximately ±0.5 °C). A proportional-integral controller provided looped feedback to maintain a stable temperature for each plate. The s.d. in the temperature at either plate over a 15 min period was 0.03 °C. The temperature gradient was confirmed to be linear vertically throughout the cell (see Supplementary Fig. 6). To do this, the mean squared displacement of vesicles in each slice was found either using particle tracking of vesicles in the *xy* plane, or using differential dynamic microscopy^41^, and used to calculate the diffusion coefficient at that vertical height; the two methods gave the same result. Then, assuming spherical objects and the Stokes–Einstein diffusion coefficient, temperature was calculated for each slice in the *z*-stack, accounting for the temperature dependence of the viscosity of the surrounding water. We also verified the wall temperature internal to the cell matches (to the precision of this approach) the measurements of the thermocouples placed on the outer walls of the sapphire chamber. We can therefore rule out any measurable temperature drop across the thin and highly thermally conductive sapphire windows.

The sample cell consisted of a 200 μm spacer cut from silicone sheet (Silex Silicones Ltd.), which was sandwiched between two sapphire windows (32 × 37.5 × 0.75 mm^3^) to form an air-tight seal. The windows were loaded between two metal plates (64 × 54 × 8 mm^3^) with holes cut for the objective lens. The sapphire windows were cleaned by boiling in 2%v Hellmanex III solution (Hellma, a commercially available detergent), followed by sonication for 15 min. The windows were then rinsed thoroughly with MilliQ water and re-sonicated for another 15 min before drying in nitrogen.

Fluorescence imaging of the vesicles was carried out using a Nikon Eclipse Ti-E inverted microscope equipped with a 40 × objective lens (Nikon, S Plan Fluor, ELWD 2.8–3.6 mm, NA 0.6) and a camera (Point Grey Research, Grasshopper3 GS3-U3-23S6M). Using standard optical methods^42^ and the parameters of the objective lens, we have calculated the depth of focus as approximately ±1.58 μm. Samples were illuminated with either a metal halide lamp or a single-colour light-emitting diode in conjunction with a Texas Red filter set (Semrock, exciter FF01-562/40, dichroic FF593-Di02, emitter FF01-624/40) or a green fluorescent protein filter set (Semrock, exciter FF01-472/30, dichroic FF495-Di03, emitter FF01-520/35).

A *z*-stack was taken through the cell (with slices 8 μm apart) and each slice was processed using custom-made MATLAB image analysis routines: A bandpass filter was applied to each slice before converting the image to binary, and then the area fraction of vesicles (white pixels/total pixels) was extracted for each slice (see Fig. 1b,c). We fit the logarithm of the concentration profile throughout the cell with a linear fit (see example in Fig. 1d) and knowing the thermal gradient we use equation (2) to calculate the Soret coefficient. An example of the vesicle area fraction at the hot and cold plates is given with respect to time in Supplementary Fig. 7 into the steady state. Vesicles are free to diffuse horizontally and are not attached to the plates. Note that the same values for the Soret coefficients were obtained using vesicle concentrations acquired by counting the number of distinct vesicles in a slice (Supplementary Fig. 8) and that the decrease in fluorescence intensity with increasing temperature was not significant enough to prevent use of thresholding in determining the area fraction of vesicles in each slice (Supplementary Fig. 9). To calculate the thermophoretic mobility, we evaluate *D* using the Stokes–Einstein equation, using the known vesicle radius, temperature (taken as 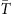) and viscosity (at 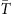), then we use equation (3) to calculate *D*_T_ (at 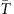).

### Data availability

A complete data set is available for download at https://doi.org/10.17863/CAM.8120.

## Additional information

**How to cite this article:** Talbot, E. L. *et al*. Thermophoretic migration of vesicles depends on mean temperature and head group chemistry. *Nat. Commun.* **8,** 15351 doi: 10.1038/ncomms15351 (2017).

**Publisher’s note**: Springer Nature remains neutral with regard to jurisdictional claims in published maps and institutional affiliations.

## Supplementary Material

Supplementary Information Supplementary Figures and Supplementary Table

## Figures and Tables

**Table 1:** Fitting parameters to equation (4).

**Lipid**	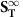 **/K**^**−1**^	T*/K	***T***_**0**_**/K**	***dD***_**T**_**/*****d***_**T**_**/10**^**−13**^ **m**^**2**^ **s**^**−1**^ **K**^**−2**^	***T*** **(*****D***_**T**_**=0)**
DOPC (○)	0.27	9.4	21.8	0.051	10.4
DiPhyPC (◊)	0.27	12.5	11.6	0.060	11.6
DPPC (▪)	0.60	16.9	36.2	0.062	17.7
DMPC (★)	0.67	16.3	36.2	0.028	14.2
DGDG (▴)	0.15	16.7	27.7	0.028	17.4
SQDG (◃)	0.20	24.4	17.0	0.054	24.5
POPG (▿)	—	—	—	−0.007	—
DOPG/DOPC/chol (○), *T*<35 °C	—	—	—	0.11	24.2
DOPG/SQDG/chol (◃), *T*<35 °C	—	—	—	0.10	28.4

chol, cholestanol; DGDG, digalactosyl diacylglycerol; DiPhyPC, 1,2-diphytanoyl-sn-glycero-phosphatidylcholine; DMPC, 1,2-dimyristoyl-sn-glycero-3- phosphocholine; DOPC, 1,2-dioleoyl-sn-glycero-3-phosphocholine; DOPG, 1,2-dioleoyl-sn-glycero-3-phospho-(1′-rac-glycerol) (sodium salt); DPPC, dipalmitoylphosphatidylcholine; POPG, 1-palmitoyl-2-oleoyl-sn-glycero-3-phospho-(1′-rac-glycerol) (sodium salt); SQDG, sulfoquinovosyldiacylglycerol.

The parameters are for various lipids grouped by head group type as well as gradients for linear fits to the thermophoretic mobility with increasing temperature and corresponding values of *T* when *D*_T_=0 from the fits in Fig. 4. Marker shape and colours used in the Figs 3, 4, 5, 6, 7 are shown by each lipid name. Note that *D*_T_ for POPG is invariant in temperature within error.
