# Supplementary material for: Thermophoretic migration of vesicles depends on mean temperature and head group chemistry
Source: Nat Commun. 2017 May 17;8:15351. doi: 10.1038/ncomms15351 (PMC5512737; doi:10.1038/ncomms15351)
Supplement: Supplementary Information — Supplementary Figures and Supplementary Table [file ncomms15351-s1.pdf]

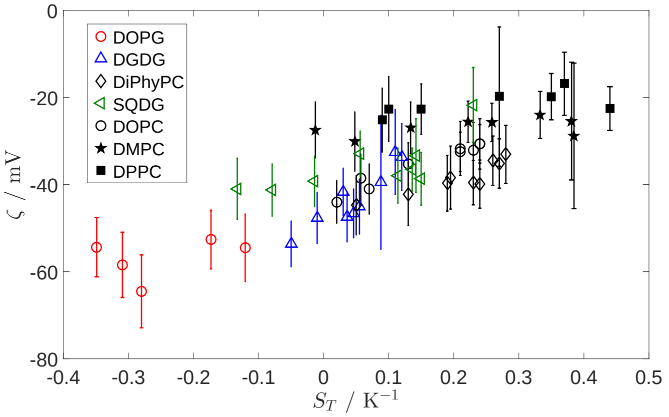

Supplementary Figure 1. **The Soret coefficient increases as the zeta potential of the vesicles becomes more negative when considering all lipid types**. However, within each lipid type there is no correlation. Error bars indicate the standard deviation.

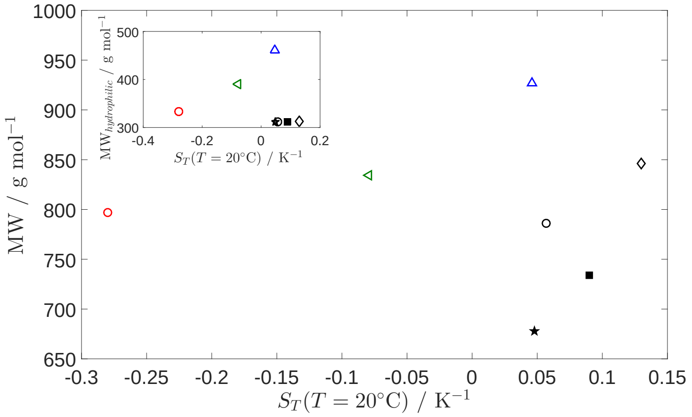

Supplementary Figure 2. **Variation of the Soret coefficient, S_T with molecular weight for each lipid type at 20°C**. The insert shows the molecular weight of only the hydrophilic headgroup portion of the lipid.

Lipid	a	b	c
DOPC* (cold side, Fig. 5a)	-1.68	0.047	5.32
POPG* (cold side, Fig. 5a)	-0.08	0.00	1.45
DOPC (hot side, Fig. 5a)	0.23	0.56	0.20
POPG* (hot side, Fig. 5a)	-1.49	0.096	5.52
DOPC (cold side, Fig. 5b)	1.00	0.003	0.1
DGDG (cold side, Fig. 5b)	1.09	0.004	0.00
DOPC (hot side, Fig. 5b)	0.87	0.001	0.00
DGDG* (hot side, Fig. 5b)	2.76	-7.1×10^{-4}	-6.11
DOPC* (cold side, Fig. 5c)	4.9	-5.3	27.5
DGDG* (cold side, Fig. 5c)	3.7	-5.0	17.8
DOPC (hot side, Fig. 5c)	0.99	0.23	0.008
DGDG (hot side, Fig. 5c)	0.98	0.18	0.017

Supplementary Table 1. **Fitting parameters to the data in figure 5 for the segregation of vesicles composed of different head-groups with time**. Fits are based on $A/A_{c0} = a[1 - \exp((b - t)/c)]$ for exponetial increases in area fraction with time (lipids marked with *), and $A/A_{c0} = a\exp(1 - bt) + c$ for exponetial decreases in area fraction with time.

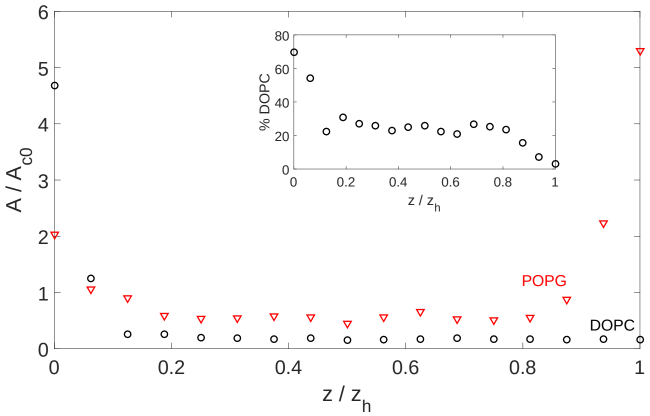

Supplementary Figure 3. **Thermophoretic migration of a 1:1 binary mixture of POPG and DOPC vesicles**. Vesicles are $1\,\mu$m in diameter with $T = 25°$C at the cold plate and $T = 55°$C at the hot plate. The area fraction of each vesicle population at the cold plate, A, is shown with time normalised by the initial area fraction at the cold plate, A_{c0}. We attribute the slight increase in POPG (triangles) concentration on the cold side to the attraction to the oppositely charged sapphire window. The overall migratory behaviour of POPG is thermophilic. The insert shows the segregation of DOPC (circles) relative to POPG as a percentage throughout vertical distance z in the cell, where z_h is the hot side of the cell.

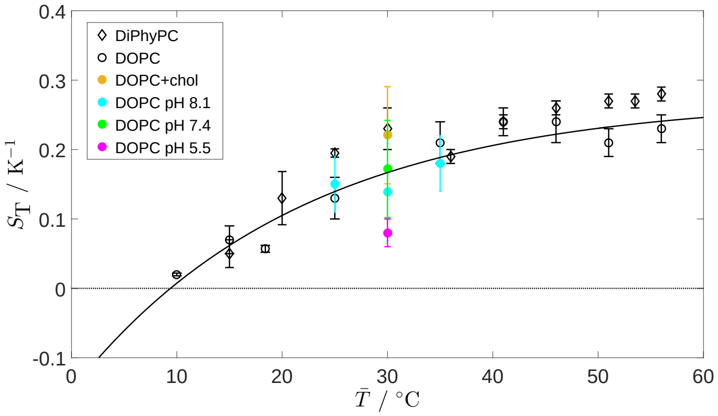

Supplementary Figure 4. **The pH causes variation in the Soret coefficient**. Soret coefficients, S_{T},are shown with mean temperature, $\bar{T}$, for DOPC vesicles in buffer at different pH and for DOPC vesicles containing 45%mol chol. Error bars on data indicate the standard deviation of S_{T} from five measurements taken at 5 minute time intervals after achieving steady state.

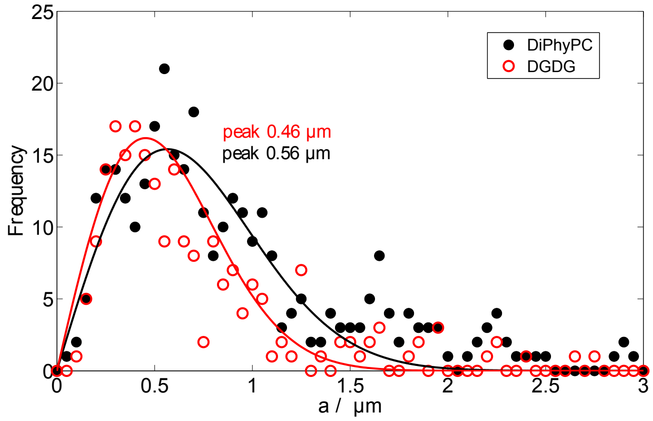

Supplementary Figure 5. **Typical size distribution for extruded vesicles**. All vesicles were formed by extrusion of different lipids through pores of 1 μm diameter. The peak frequency is at a vesicle radius, a, of $\sim$0.56 μm. Vesicle radii were determined based on their Brownian motion (at a known temperature) using particle tracking algorithms. Frequencies are for size bins of 0.05 μm.

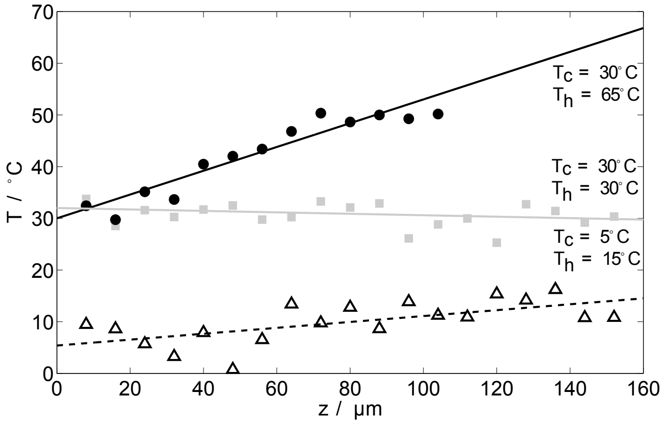

Supplementary Figure 6. **The temperature distribution vertically throughout the cell**. Temperatures were calculated at steady state based on diffusion coefficients of DGDG vesicles. Mean squared displacements of the vesicles were found in each slice using a particle tracking algorithm. From these displacements, the diffusion coefficient was obtained for that slice. Then, assuming spherical vesicles and a Stokes-Einstein relationship, the temperature of that slice was calculated accounting for the temperature dependence of the viscosity. The temperature of the hot plate, $T_{\rm h}$, and the cold plate, $T_{\rm c}$, are indicated. As the data were collected at steady state, thermophoretic migration of vesicles to the cold side limited data collection at large z for the $T_{\rm h}$=65°C dataset. Therefore, data points for $T_{\rm h}$=65°C cover only about two thirds of the height as the cell is depleted of vesicles elsewhere: we cant measure the mean squared displacement and generate temperature data here. Lines are linear fits.

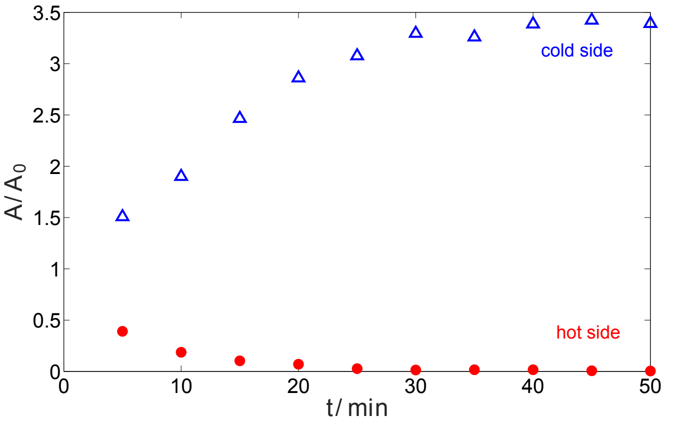

Supplementary Figure 7. **The area fraction of DGDG vesicles on the hot and cold side of the cell with time**. The area fraction, A, is normalised by the initial area fraction on that side, A_0.

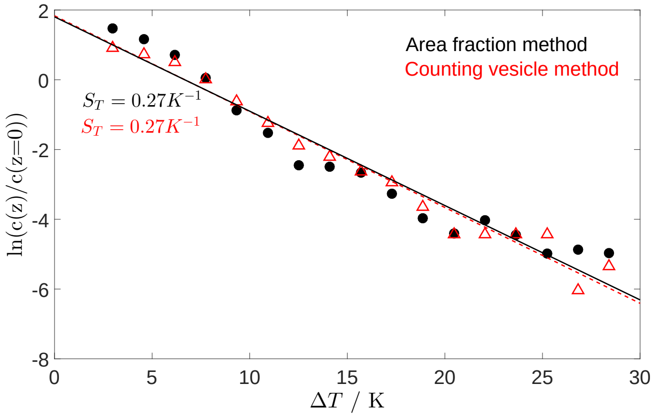

Supplementary Figure 8. **Comparison between area fraction analysis and counting vesicle method for obtaining the Soret coefficient**. Both methods give the same result. The example here is for DiPhyPC vesicles with T_c=36°C and T_h=66°C.

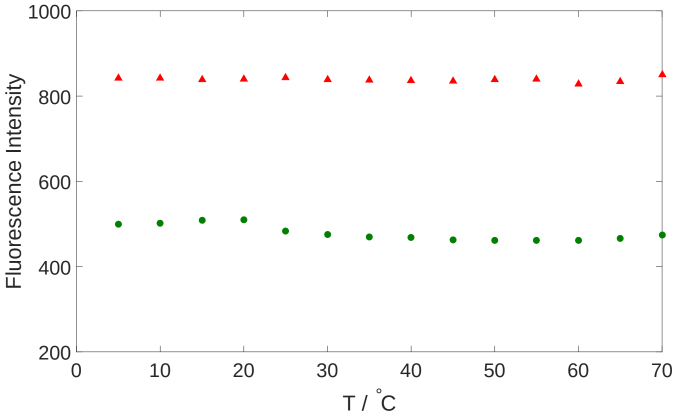

Supplementary Figure 9. **Fluorescence intensity variance with temperature for DOPC vesicles**. Vesicles contain 0.8 mol% of the fluorescent lipids DHPE-TX (red) or DHPE-OG (green).
